# Pituitary Transcriptomic Study Reveals the Differential Regulation of lncRNAs and mRNAs Related to Prolificacy in Different FecB Genotyping Sheep

**DOI:** 10.3390/genes10020157

**Published:** 2019-02-18

**Authors:** Jian Zheng, Zhibo Wang, Hua Yang, Xiaolei Yao, Pengcheng Yang, CaiFang Ren, Feng Wang, YanLi Zhang

**Affiliations:** 1 Jiangsu Engineering Technology Research Center of Mutton Sheep and Goat Industry, Nanjing Agricultural University, Nanjing 210095, China; 2016105032@njau.edu.cn (J.Z.); 2018105081@njau.edu.cn (Z.W.); 2018205011@njau.edu.cn (H.Y.); 2016205004@njau.edu.cn (X.Y.); rencaifang@hotmail.com (C.R.); caeet@njau.edu.cn (F.W.); 2 Jiangsu Livestock Embryo Engineering Laboratory, Nanjing Agricultural University, Nanjing 210095, China; 3 National Experimental Teaching Demonstration Center of Animal Science, Nanjing Agricultural University, Nanjing 210095, China; 2017805111@njau.edu.cn

**Keywords:** lncRNA, pituitary function, RNA-Seq, Hu sheep

## Abstract

Long non-coding RNA (LncRNA) have been identified as important regulators in the hypothalamic-pituitary-ovarian axis associated with sheep prolificacy. However, their expression pattern and potential roles in the pituitary are yet unclear. To explore the potential mRNAs and lncRNAs that regulate the expression of the genes involved in sheep prolificacy, we used stranded specific RNA-seq to profile the pituitary transcriptome (lncRNA and mRNA) in high prolificacy (genotype *FecB* BB, litter size = 3; H) and low prolificacy sheep (genotype *FecB* B+; litter size = 1; L). Our results showed that 57 differentially expressed (DE) lncRNAs and 298 DE mRNAs were found in the pituitary between the two groups. The qRT-PCR results correlated well with the RNA-seq results. Moreover, functional annotation analysis showed that the target genes of the DE lncRNAs were significantly enriched in pituitary function, hormone-related pathways as well as response to stimulus and some other terms related to reproduction. Furthermore, a co-expression network of lncRNAs and target genes was constructed and reproduction related genes such as *SMAD2*, *NMB* and *EFNB3* were included. Lastly, the interaction of candidate lncRNA MSTRG.259847.2 and its target gene *SMAD2* were validated in vitro of sheep pituitary cells. These differential mRNA and lncRNA expression profiles provide a valuable resource for understanding the molecular mechanisms underlying Hu sheep prolificacy.

## 1. Introduction

Hu sheep are a Chinese indigenous breed with high prolificacy and year-round estrus. It is important to investigate the genes involved in its high prolificacy traits. To date, mutations in *BMP15*, *GDF9* and *BMPR-1B* have been found in some sheep breeds as fecundity genes that affect follicular development and ovulation. Although existing genetic studies have already identified some sheep fecundity genes, the underlying genetic mechanisms remain largely unknown. *FecB* is a key candidate gene for the genetic control of sheep reproductive performance, which is known as the first major gene associated with sheep prolificacy [1,2]. It has been found in Booroola Merino sheep [3], Javanese Indonesia sheep [4], Small Tailed Han sheep [5], Garole [6], Kendrapada [6] and also Hu sheep [5]. Recent studies have shown that *FecB* gene has close relationship with litter size of Hu sheep and the frequency of the *FecB* allele in Hu sheep is up to 53% [7]. Therefore, we selected Hu sheep with different fecundity according to *FecB* genotyping in our study as experimental material.

In recent years, long non-coding RNA (lncRNA) has attracted much attention, which is a class of non-coding RNA with a length of more than 200 nucleotides [8]. LncRNAs regulate gene expression through epigenetic regulation, transcription and post transcriptional regulation [9] and are involved in many biological processes such as cell proliferation and differentiation [10], ontogeny [11], signal transduction [12] and stem cell maintenance [13]. Some studies have also found that lncRNAs involve in Gonadgenesis [14], Sex determination [15], Sex hormone responses [16], Meiosis [17], Spermatogenesis [12] and Placentation [18,19]. Furthermore, lncRNA have been identified as important regulators in the hypothalamic-pituitary-ovarian (HPO) axis associated with reproduction. However, their expression pattern and potential roles in the pituitary are not yet clear. Previous studies of the pituitary mainly focus on miRNAs and mRNA [20,21,22,23], limited lncRNA studies were reported on rats [24] or related to pituitary tumorigenesis [25,26]. Han et al. (2017) recently reported that immature and mature rats had different lncRNAs in the anterior pituitary [24]. The HPO axis is one of the determinants of the fecundity. The pituitary is not only regulated by the hypothalamus but also influences the ovarian function through hormones and other regulatory factors, which play a connecting role in the HPO axis. When abnormal gene modifications occur in the pituitary, they probably pass through the HPO axis then affect fecundity of domestic animals [27]. However, a systematic analysis of lncRNAs expressed at normal pituitary related with different fecundity, particularly in sheep, has not been performed.

In this study, to identify the role of lncRNAs and mRNAs in the pituitary associated with different sheep prolificacy based on *FecB* genotyping (*FecB* BB, litter size = 3. versus *FecB* B+; litter size = 1). The target genes of the DE lncRNAs and the DE genes (DEGs) were examined. DE lncRNAs were then used bioinformatics analysis to predict cis- and trans-target genes. Most importantly, the interaction of candidate lncRNA MSTRG.259847.2 and its target gene *SMAD2* were validated in vitro of sheep pituitary cells. This study expands the lncRNA catalogue in sheep pituitary and provides candidate regulators of sheep prolificacy at the transcriptional level.

## 2. Materials and Methods

### 2.1. Animals and Sample Collection

All related experiments involving sheep were conducted in strict compliance with relevant guidelines set by the Ethics Committee of Nanjing Agricultural University, China (Approval ID: SYXK2011-0036).

Sheep used in this study were raised under the same conditions at Taizhou Hailun Sheep Industry Co., Ltd. (Taizhou, China). A total of 6 non-pregnant ewes with identical lambing records (3 records) were selected and divided into a high prolificacy group (H: *n* = 3, genotype *FecB* BB, litter size = 3) and a low prolificacy group (L: *n* = 3, genotype *FecB* B +, litter size = 1). Synchronous estrus were conducted before the experiment, the vaginal sponge (CIDR) was implanted for 11 days, followed by the administration of 100 IU PG at the time of sponge removal. Estrus condition of the ewes was monitored three times one day and slaughtered at the second natural estrus within 12 h. After slaughtering, pituitary samples were immediately collected and stored at −80 °C for total RNA extraction.

### 2.2. RNA Extraction and Library Preparation

TRIzol reagent (Invitrogen, California, USA) with DNase I (Qiagen, Beijing, China) was used to extract the total RNA of the pituitary and monitored on 1.5% agarose gels. RNA concentration and purity were measured using the NanoDrop 2000 Spectrophotometer (Thermo Fisher Scientific, Wilmington, DE, USA). RNA integrity was assessed using the RNA Nano 6000 Assay Kit of the Agilent Bioanalyzer 2100 System (Agilent Technologies, Santa Rosa, CA, USA).

A total amount of 1.5 μg RNA per sample was used as input material for rRNA removal using the Ribo-Zero rRNA Removal Kit (Epicentre, Madison, WI, USA). Sequencing libraries were generated using NEBNext UltraTM Directional RNA Library Prep Kit for IlluminaR (NEB, Ipswich, MA, USA) following the manufacturer’s instructions. Briefly, fragmentation was carried out using divalent cations under elevated temperature in NEBNext First Strand Synthesis Reaction Buffer (5×). First strand cDNA was synthesized using random hexamer primer and reverse transcriptase. Second-strand cDNA synthesis was subsequently performed using DNA Polymerase I and RNase H. Remaining overhangs were converted into blunt ends via exonuclease/polymerase activities. After adenylation of 3′ ends of DNA fragments, NEBNext Adaptor with hairpin loop structure were ligated to prepare for hybridization. In order to preferentially select fragments with 150~200 bp in length, the library fragments were purified with AMPure XP Beads (Beckman Coulter, Beverly, MA, USA). Then 3 μL USER Enzyme (NEB, Ipswich, MA, USA) was used for size-selected, adaptor-ligated cDNA at 37 °C for 15 min. Then PCR was performed with Phusion High-Fidelity DNA polymerase, universal PCR primers and Index (X) Primer. Finally, PCR products were purified (AMPure XP system) and library quality was assessed on the Agilent Bioanalyzer 2100 and qPCR.

### 2.3. Clustering, Sequencing and Transcriptome Assembly

The raw data were first filtered to remove low-quality reads by passed in-house perl scripts, then the clean data, through repeated testing, were assembled using the HISAT2 and String Tie based on the reads mapping to the reference genome (Ovis aries v4.0). The assembled transcripts were annotated using the gffcompare program. The unknown transcripts were used to screen putative lncRNAs. Putative protein-coding RNAs were filtered out based on a minimum length and exon number threshold. Transcripts with lengths more than 200 nt and two exons were selected as lncRNA candidates. Next, they were screened using CPC (Coding-Non-Coding Index)/CNCI (Coding-Non-Coding Index)/Pfam (protein families database)/CPAT (Coding Potential Assessment Tool) to distinguish the protein-coding genes from the non-coding genes. By comparing transcripts with known protein databases, CPC is used to assess the coding potential of transcripts based on their biological sequence characteristics. When Score < 0, it is regarded as non-coding RNA [28]. CNCI analysis is a method to distinguish coding transcripts from non-coding transcripts by the characteristics of adjacent nucleotide triplets. When score < 0, it is regarded as non-coding RNA [29]. CPAT analysis is another method of judging encoding and non-encoding ability of transcript by constructing logistic regression model, which calculate Fickett score and Hexamer score based on open reading frame (ORF) length and ORF coverage (*E*-value < 0.001) [30]. Pfam divides protein domains into different protein families and establishes HMM statistical models of amino acid sequences of each family by comparing protein sequences. The transcripts that can be compared are those with a protein domain which coding ability, while those with no comparable results are potential lncRNAs [31]. LncRNA for subsequent analysis was obtained by intersecting the above four analysis results. The different types of lncRNAs including lincRNA, intronic lncRNA, anti-sense lncRNA and sense lncRNA were identified using Cuffcompare.

### 2.4. Differential Expression Analysis and qRT-PCR Validation

Fragments Per Kilobase of transcript per Million fragments mapped (FPKM) is used as an indicator to measure transcript or gene expression levels. StringTie (1.3.1) (https://ccb.jhu.edu/software/stringtie/index.shtml) was used to calculate FPKMs of both lncRNAs and protein coding genes in each sample. Genes with an adjusted *p*-value < 0.05 and absolute value of log2(Fold change) > 1 found by DESeq [32] were assigned as differentially expressed. Log2FC was calculated based on standardized counts and has a strong correlation with FPKM value. The hierarchical clustering analysis of the DE lncRNA and mRNA are made to cluster the lncRNA with similar expression characteristics.

Primers of randomly selected genes were designed and synthesized by the public Biotech Corp (Nanjing, China). For the qRT-PCR analysis, 1 μg total RNA was reverse transcribed using the RT reagent Kits with gDNA Eraser (Takara, Beijing, China) according to the manufacturer’s protocol. qRT-PCR was performed on a StepOnePlus Real-Time PCR System (Life Technologies, NY, NY, USA) according to the standard methods using Fast Start Universal SYBR Green Master (ROX) (Roche, Basel, Switzerland). Six DE lncRNAs and six DEGs were randomly chosen for validation. Comparative quantification of each gene was normalized to hypoxanthine phosphoribosyl transferase 1(*HPRT1*) using the 2^−ΔΔCt^ method. All experiments were performed in triplicate. The primers are listed in Table S1.

### 2.5. Target Gene Prediction and Functional Annotation Analysis

Prediction of DE lncRNAs target genes by cis-and trans-acting. For each lncRNA locus, the 100 kb downstream and upstream protein-coding genes (without overlap) were firstly identified as cis-acting target genes. Then, the genes that overlapped with the lncRNAs predicted by Lnctar (http://www.cuilab.cn/lnctar) were selected as the trans-acting target genes.

Gene Ontology (GO) enrichment analysis of the DEGs was implemented by the topGO R packages. We used KOBAS [33] software (KOBAS 3.0, http://kobas.cbi.pku.edu.cn/index.php, USA) to test the statistical enrichment of DE genes in Kyoto Encyclopedia of Genes and Genomes (KEGG) pathways. The sequences of the DEGs were blast (blastx) to the genome of a related species (the protein-protein interaction (PPI) of which exists in the STRING database: http://string-db.org/) to obtain the predicted PPI of these DEGs.

### 2.6. Construction of the LncRNA-gene Co-Expression Network

To further explore the interactions between the lncRNAs, target genes and DEGs in female reproduction. Based on the Pearson correlation index calculation between mRNA and lncRNA, we screened the most related lncRNAs and their targeted genes. Then the LncRNA-target gene-DEGs is built with PPI. The networks associated with pituitary function and reproduction were sorted with reference to their GO and KEGG enriched terms with key words. Visualization of gene interaction is achieved through an open software platform called Cytoscape (Cystoscape 3.7.1, https://cytoscape.org/, USA) [34].

### 2.7. Pituitary Cells Isolation, Transfection and qRT-PCR Validation

Primary pituitary cells were isolated from 2M ewes pituitary using collagenase IV (0.1%, 15 min) and Trypsin (0.25%, 15 min) in the lab and cultured in DMEM/F12 (Gibco Life Technologies, Carlsbad, CA, USA), supplemented with 10% FBS (Gibco Life Technologies, NY, NY, USA), 100 U/mL penicillin, at 37 °C in a 5% CO_2_ atmosphere. The siRNAs of lncRNA MSTRG.259847.2 were synthesized by the GenePharma company, Shanghai, China. The sequences of three siRNAs are listed in Table S1.

Next, the siRNAs were transfected into sheep pituitary cells using the Lipofectamine 3000 reagent (Invitrogen Life Technologies, Carlsbad, CA, USA), according to the manufacturer’s protocol. The cells were harvested for qPCR after incubation for 30 h. The expression levels of MSTRG.259847.2 and its targeted gene *SMAD2* were analyzed by qRT-PCR.

### 2.8. Immunofluorescence Staining of Primary Pituitary Cells

Immunofluorescence staining was performed, following previously described methods [35]. The rabbit anti- Luteinizing Hormone (LH, 1:200, Bioss, bs-0952R; Beijing, China,) and Follicle-Stimulating Hormone (FSH, 1:200, Bioss, bs-1536R; Beijing, China,) were used as the primary antibody and 594-conjugated Donkey Anti-Rabbit antibody was used as the secondary antibody (1:200, Abcam, #Ab96921; Cambridge Science Park, UK). Nuclei were stained with 4, 6-diamidino-2-phenylindole (DAPI). The negative controls were not incubated with the primary antibody and only treated with the secondary antibody. Images were obtained using a fluorescence microscope (Nikon, Tokyo Met, Japan).

### 2.9. Statistical Analysis

Each group had three samples and all the experiments were repeated at least three times. The data were analyzed using SPSS [36] software (20.0 Edition, Chicago, IL, USA). The results represent a mean value of ± standard error, the difference between data is analyzed using a t test (*p* < 0.05).

## 3. Results

### 3.1. Overview of Sequencing Data in Sheep Pituitary Tissue

In this study, a total of 130.81 Gb of clean data were obtained. The clean data of each sample reached 17.74 Gb and the percentage of Q30 base was higher than 91.37% which indicated that the sequencing data was highly reliable (Table S2). The average reads number of the six samples reached 145,780,632, the ratio of mapped reads and unique mapped reads ratio were 91.30% and 80.42% respectively and the ratio of multiple mapped reads was less than 11.19% (Table S2).

### 3.2. Identification of LncRNAs and mRNAs in Hu Sheep Pituitary Tissue

**Figure 1:**
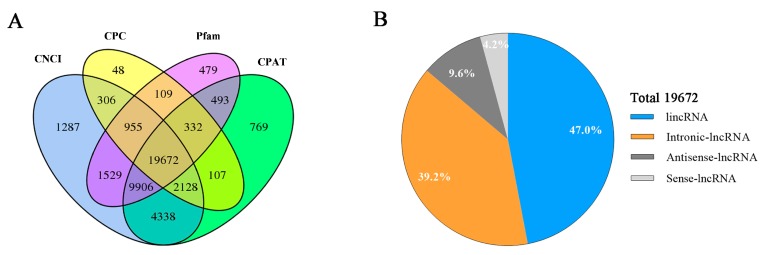
Identification of lncRNAs. (**A**) The lncRNAs were identified from the intersection of CNCI, CPC, Pfam and CPAT. (**B**) Classification of 19,672 lncRNAs, including LincRNAs, Intronic-lncRNAs, Antisense-lncRNAs and Sense-lncRNAs.

**Figure 2:**
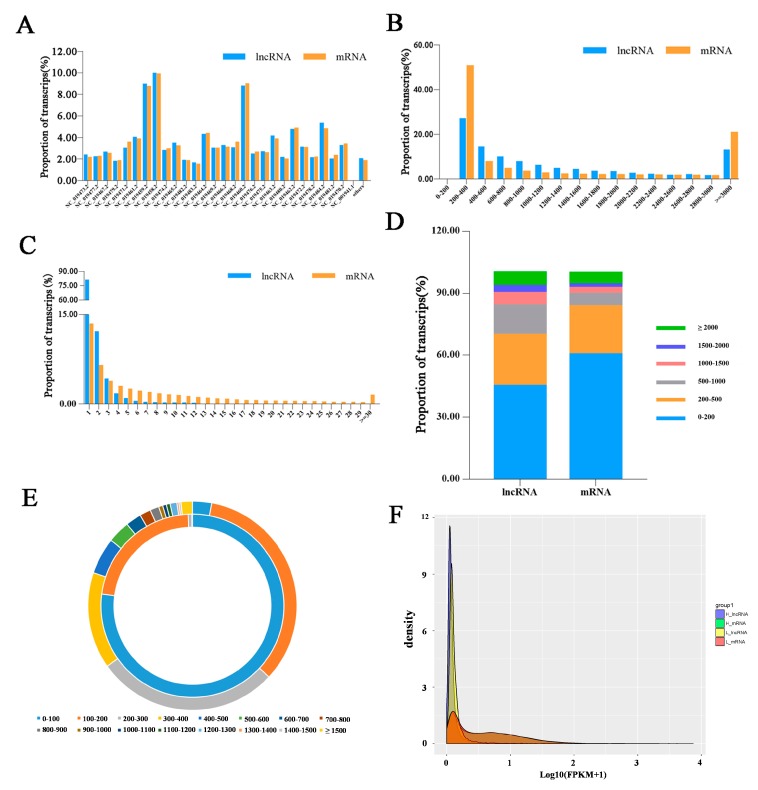
Identification of lncRNAs and protein-coding genes in Hu sheep pituitary. (**A**) Distribution of lncRNAs and protein-coding genes in chromosomes. (**B**) Length of lncRNAs and protein-coding genes. (**C**) Exon content of lncRNAs and protein-coding genes. (**D**) Exon size distribution of sheep lncRNAs and protein-coding genes. (**E**) Length of the open reading frame (ORF) of lncRNAs and protein-coding genes. (**F**) The Fragments Per Kilobase of transcript per Million fragments mapped (FPKM) levels of lncRNA and protein-coding genes in the H and L group.

After mapping to the reference sequence, the results of CPC/CNCI/Pfam/CPAT software were combined to screen lncRNAs (Figure 1A). We identified 19,672 lncRNAs, including 9237 lincRNAs (47.0%), 7720 intronic lncRNAs (39.2%), 1,879 antisense lncRNAs (9.6%) and 836 sense lncRNAs (4.2%) (Figure 1B). These lncRNAs and mRNAs were randomly assigned to 26 autosomes and the X-chromosome. As shown in Figure 2A, about 2.0% and 1.8% genes were not matched any chromosomal location and no lncRNAs was found in the mitochondria.

The distribution of lncRNAs and mRNAs length in the pituitary are similar. The lncRNAs and mRNAs transcripts were mainly distributed from 200 to 600 bp and the ratio of lncRNA and mRNA transcripts were decreased because of length increasing. (Figure 2B). In addition, the lncRNAs and mRNAs transcripts mainly containing 1 to 3 exons and the ratio of lncRNAs and mRNAs transcripts decreased with increasing number of exons (Figure 2C). However, the exon size of genes was smaller than that of lncRNAs (Figure 2D). In addition, the average open reading frame (ORF) length of the lncRNA transcripts (about 76 bp, on average) was shorter than that of the mRNA transcripts (about 333 bp, on average, Figure 2E). Most lncRNA transcripts were expressed at a lower level than 1 FPKM and the number of lncRNA transcripts decreased with increased FPKM. The results suggested that the number of lncRNA transcripts in the low expression region is higher than that in the mRNA transcripts (log10(FPKM + 1) < 0.5) but lower than that in the high expression region (log10(FPKM + 1)> 0.5). (Figure 2F).

### 3.3. The Profiling and Verification of DE LncRNA and DEGs of Sheep Pituitary

**Figure 3:**
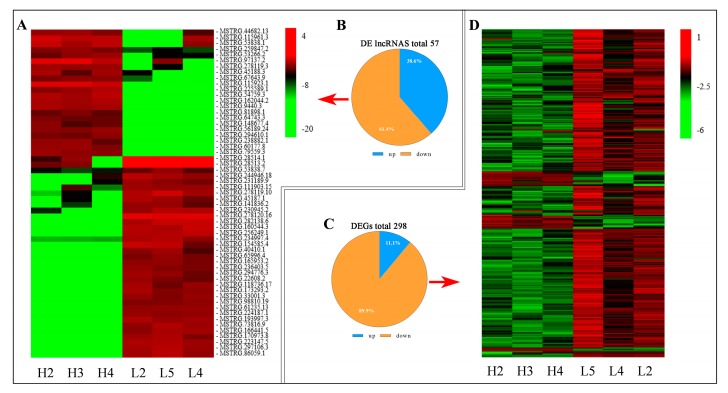
The number of differentially expressed (DE) lncRNAs and DE genes (DEGs) in H and L groups comparisons. (**A**) The hierarchical cluster of DE lncRNAs. (**B**) Total number of up-regulated and down-regulated DE lncRNAs in each comparison. (**C**) Total number of up-regulated and down-regulated DEGs in each comparison. (**D**) The hierarchical cluster of DEGs.

**Figure 4:**
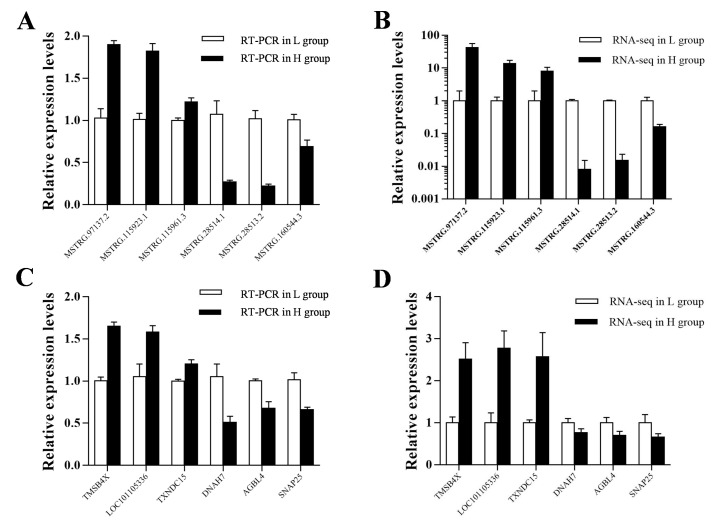
The verification of expression level of DE lncRNAs and DE mRNAs in different groups. (**A**, **B**) The relative expression level of 6 DE mRNAs in different groups determined by qRT-PCR and RNA-Seq, respectively. *TMSB4X*, thymosin β 4 X-linked; TXNDC15, thioredoxin domain containing 15; *DNAH7,* dynein axonemal heavy chain 7; *AGBL*4, ATP/GTP binding protein like 4; *SNAP25,* synaptosome associated protein 25. (**C**, **D**) The relative expression level of 6 DE lncRNAs in 2 groups determined by qRT-PCR and RNA-Seq, respectively. The relative expression level of DE lncRNAs and DE mRNAs in the pituitary was determined by qRT-PCR and normalized to the expression amount of *HPRT1*. The qRT-PCR data were represented as the mean ± SEM of three biological and technical replicates. The RNA-Seq data represented the FPKMs of each transcript.

In total, we identified 57 DE lncRNA transcripts (Figure 3B) and 298 DE mRNA transcripts (Figure 3C) between the two groups following criteria of the false discovery rate (FDR) < 0.05 and the fold change (FC) > 2. The systematic clustering analysis was used to compare the expression patterns of DE lncRNAs (Figure 3A) and DEGs (Figure 3D) for searching similarities and differences. For further evaluating the results of RNA sequencing, six DE lncRNAs (MSTRG.97137.2, MSTRG.115953.1, MSTRG115961.3, MSTRG.28514.1, MSTRG.28513.2 and MSTRG.160544.3) and protein-coding gene (*TMSB4X*, LOC101105336, *TXNDC15*, *DNAH7*, *ACBL4* and *SNAP25*) transcripts were randomly selected and their expression levels in H and L groups were verified using qRT-PCR. The results confirmed by the expression levels of the six lncRNAs and protein-coding gene transcripts were consistent with the RNA-seq results (Figure 4).

### 3.4. GO and KEGG Analysis of DEGs

We performed GO and KEGG enrichment analysis on 33 up-regulated genes and 265 down-regulated genes. Among them, 27 up-regulated and 159 down-regulated genes were annotated by GO enrichment analysis. As shown in Table S3, the top 20 most significant enriched GO terms involved include single organismal cell-cell adhesion, RNA-dependent DNA replication, cellular respiration, positive regulation of spermidine biosynthetic process, putrescine biosynthetic process and so forth. Furthermore, several GO terms which related to pituitary function and reproduction were enriched, including reproduction, reproductive process, response to stimulus, synapse (Table S4).

**Figure 5:**
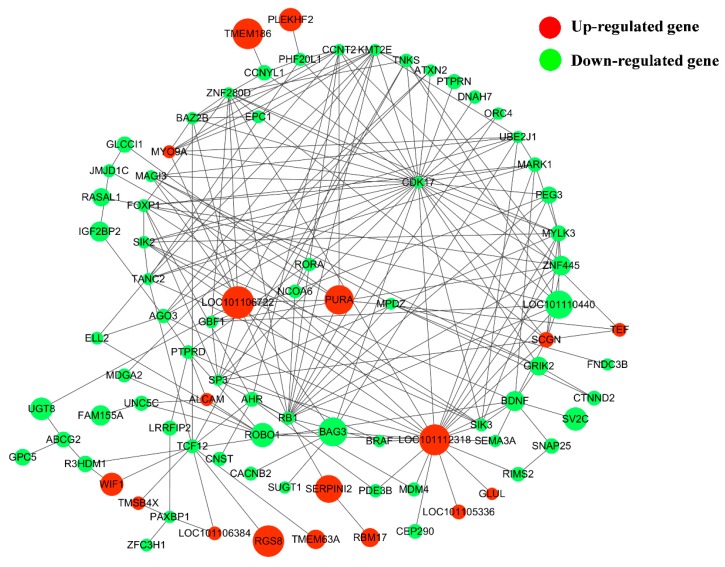
The network of 87 screened DE genes enriched for Hu sheep pituitary functions and reproduction related pathways were constructed, the red and green circular represent upregulated and downregulated DE genes, respectively. Node size represents the fold change of a node. Information about genes is shown in Table S6.

Based on KEGG enrichment analysis, 21 up-regulated genes and 107 down-regulated genes were annotated to 1588 pathways (Table S5). Among them, the pathway associated with pituitary function and reproduction included cAMP, NF-kappa B, TGF-β, PI3K-Akt, MAPK, Hippo, cGMP-PKG and mTOR signaling pathways, as well as several hormone-related pathways like Oxytocin, GnRH and Insulin signaling pathways. As shown in Figure 5, 82 downregulated and 22 upregulated genes were interacted with pituitary functions and reproduction in the network. The results showed that *SIK2*, *SIK3*, *SCGN*, *MARK1*, *MAGI3*, *CDK17*, *ZNF455* and several new genes such as LOC101112318, LOC101110440 were the hub genes in the interaction networks.

### 3.5. Screening of Potential Functional LncRNAs Involved in Hu Sheep Reproduction

**Figure 6:**
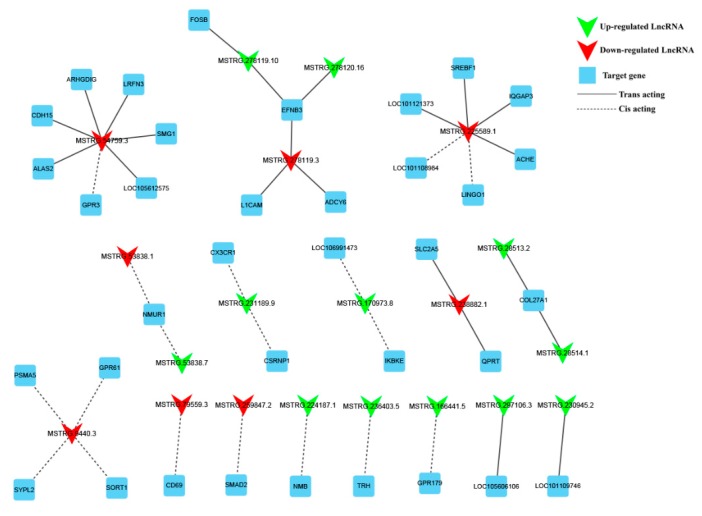
20 DE lncRNAs and their predictably interactive 36 cis- and trans-targeted genes were composed of this interactive network. The red and green color represent up- and down-regulation, quadrilaterals represent lncRNA, the box represents targeted genes, the straight line and dotted line represent the interaction relationship with trans-and cis-regulation, respectively. *CDH15*, Cadherin-15; *ARHGDIG*, Rho GDP dissociation inhibitor γ; *LRFN3*, leucine rich repeat and fibronectin type III domain containing 3; *SMG1*, suppressor of morphogenesis in genitalia 1; *ALAS2*, 5′-aminolevulinate synthase 2; *GPR3*, G protein-coupled receptor 3; *FOSB*, FosB proto-oncogene, AP-1 transcription factor subunit; *EFNB3*, ephrin B3; *L1CAM*, L1 cell adhesion molecule; *ADCY6*, adenylate cyclase 6; SREBF1, sterol regulatory element binding transcription factor 1; *IQGAP3*, IQ motif containing GTPase activating protein 3; *ACHE*, acetylcholinesterase; *LINGO1*, leucine rich repeat and Ig domain containing 1; *NMUR1*, neuromedin U receptor 1; *CXCR1*, C-X-C motif chemokine receptor 1; *CSRNP1*, cysteine and serine rich nuclear protein 1; IKBKE, inhibitor of nuclear factor kappa B kinase subunit epsilon; *SLC2A5*, solute carrier family 2 member 5; *QPRT*, quinolinate phosphoribosyl transferase; *PSMA5*, proteasome subunit α 5; *GPR61*, G protein-coupled receptor 61; SYPL2, synaptophysin like 2; *SORT1*, sortilin 1; *CD69*, CD69 molecule; *SMAD2*, SMAD family member 2; *NMB*, neuromedin B; *TRH*, thyrotropin releasing hormone; *GPR179*, G protein-coupled receptor 179.

To further explore the lncRNAs related to pituitary function and reproduction of Hu sheep, we constructed the interaction network of lncRNAs and their cis- and trans-target genes. 20 DE lncRNAs and 36 target genes are enriched in pituitary function and reproduction in Figure 6. The target genes of these DE lncRNA are enriched in GO terms including hormone secretion, reproduction, reproductive process, response to stimulus and synapse part and KEGG pathways including cAMP, ovarian steroidogenesis, estrogen and progesterone-mediated oocyte maturation signaling pathways. 18 cis-regulation and 19 trans-regulation relationships were involved in this network. MSTRG.54759.3 was cis-acting with *GPR3* and MSTRG.225589.1 was cis-acting with LOC101108984 and LINGO1 through sequence complementarity action, respectively. Meanwhile, lncRNA MSTRG.54759.3 was trans-acting with *ALS2*, *CDH15* and *SMG1*. LncRNA MSTRG.225589.1 was trans-acting with *ALS2*, *CDH15*, *ACHE* and LOC101121373 and so forth, respectively. The target gene *EFNB3* was regulated by two upregulated lncRNAs and one downregulated lncRNAs.

### 3.6. Construction of DE lncRNA-Target Gene-DEG Regulated Networks

**Figure 7:**
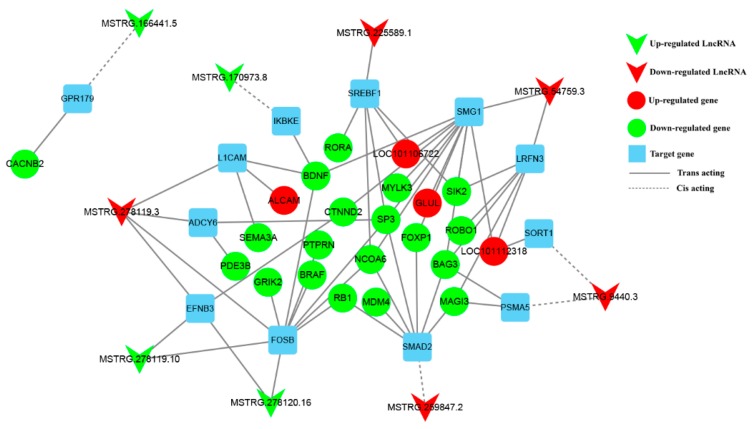
DE lncRNAs, targeted genes and DEGs were composed of the interactive network. The red and green colors represent up and down regulation, the quadrilateral, box and circles represent lncRNAs, targeted genes and DE genes, the straight line and dotted line represent the interaction relationship between trans- and cis-regulation respectively. *GPR179*, G protein-coupled receptor 179; *CACNB2*, calcium voltage-gated channel auxiliary subunit β 2; *SREBF1*, sterol regulatory element binding transcription factor 1; *SMG1*, suppressor of morphogenesis in genitalia 1; *LRFN3*, leucine rich repeat and fibronectin type III domain containing 3; *SORT1*, sortilin 1; *PSMA5*, proteasome subunit α 5; *SMAD2*, SMAD family member 2; *FOSB*, FosB proto-oncogene, AP-1 transcription factor subunit; *EFNB3*, ephrin B3; *ADCY6*, adenylate cyclase 6; *L1CAM*, L1 cell adhesion molecule; *IKBKE*, inhibitor of nuclear factor kappa B kinase subunit epsilon; *ALCAM*, activated leukocyte cell adhesion molecule; *GLUL*, glutamate-ammonia ligase; *RORA*, RAR related orphan receptor A; BDNF, brain derived neurotrophic factor; *MYLK3*, myosin light chain kinase 3; *SIK2*, salt inducible kinase 2; *CTNND2*, catenin delta 2; *SP3*, Sp3 transcription factor; *SEMA3A*, semaphorin 3A; *PTPRN*, protein tyrosine phosphatase, receptor type N; *NCOA6*, nuclear receptor coactivator 6; *FOXP1*, forkhead box P1; *ROBO1*, roundabout guidance receptor 1; *BAG3*, BCL2 associated athanogene 3; *GRIK2*, glutamate ionotropic receptor kainate type subunit 2; *BRAF*, B-Raf proto-oncogene, serine/threonine kinase; *RB1*, RB transcriptional corepressor 1; *MDM4*, MDM4, p53 regulator; *MAGI3*, membrane associated guanylate kinase, WW and PDZ domain containing 3.

Next, to further explore the functional relationship between lncRNA and DEGs, we built DE lncRNA-Target gene-DEG regulated networks in Figure 7. In total, 9 lncRNAs, 12 target genes and 23 DEGs involved were constituted two co-expression networks. The networks provide candidate lncRNAs related to pituitary function and reproduction. Furthermore, the DEGs that are directly involved in pituitary function and reproduction and their corresponding DE lncRNAs were classified. Consequently, most of the DEGs and target genes were enriched in response to stimulus, reproduction and reproductive process. In the two networks, 5 lncRNAs were trans-acting with their target genes by sequence complementarity action. Interestingly, most of the interacting genes were downregulated in H group when compared with L group.

### 3.7. Verification of MSTRG.259847.2 and Its Target Gene SMAD2 in Sheep Primary Pituitary Cells

**Figure 8:**
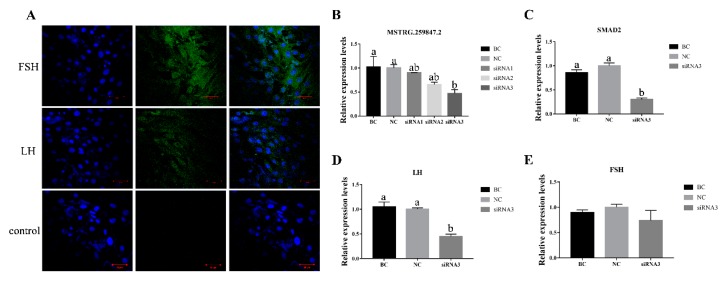
(**A**) Identification of sheep pituitary cells by FSH and LH immunofluorescence staining. The left panels DAPI staining for nucleus, middle panels show antigen-specific staining and the right panels show their merged images. Bars indicate 50μm. (**B**) The relative expression levels of lncRNA MSTRG.259847.2 in sheep pituitary cell transfecting with MSTRG.259847.2 siRNA 1, 2 and 3. (**C**) The relative expression of *SMAD2*(lncRNA MSTRG.259847.2 target gene) in sheep pituitary cell transfecting with MSTRG.259847.2 siRNA3. BC and NC represent blank and negative controls, respectively. Results are expressed relative to the BC group as mean values ± the SEM. a, b: different letters denote statistically significant differences within each group. (**D**, **E**) The relative expression of *LH* and *FSH* in transfected pituitary cells was determined using qPCR. The relative expression levels were normalized to the expression levels of *GAPDH*. Results are expressed relative to the blank control mean values ± SEM. a, b, c: different letters denote statistically significant differences within each group; *p* < 0.05.

To further validate the interaction of our screened lncRNAs and their target genes, we isolated primary sheep pituitary cells. LH and FSH, two characteristic protein marker were used to characterize these in vitro isolated pituitary cells by immunofluorescence staining. Microscopic examination showed that a large percentage cells were positive for LH and FSH (Figure 8A). Then we transfected sheep pituitary cells with lncRNA MSTRG.259847.2 siRNAs. The knock-down efficiency of siRNA3 was the highest among the three siRNAs (Figure 8B). The expression level of its target gene, *SMAD2*, in siRNA3-transfected group was significantly lower than in the control groups (Figure 8C). Furthermore, the *LH* gene expression level in lncRNA MSTRG.259847.2 knock-down group was significantly lower than that in the control groups (Figure 8D). While no significant difference was found in *FSH* expression levels among the blank control (BC), negative control (NC) and lncRNA MSTRG.259847.2 siRNA3-transfected group (Figure 8E).

## 4. Discussion

Reproduction ability has important impacts on sheep profitability. Accumulating evidence indicates the important roles of lncRNAs in sheep reproduction [37,38,39]. It is known that the pituitary secretes hormones such as LH, FSH and PRL, play crucial roles in reproductive process. However, the current studies of lncRNAs mainly focus on ovary [38,39,40]. Here, we conducted genome-wide analyses to identify mRNAs and lncRNAs that were differentially expressed in the pituitary gland of Hu sheep associated with different lambing number and *FecB* genotype. We also sought to find a relationship between lncRNAs and mRNAs by generating a co-expression network. Previous study showed that Hu sheep with genotype *FecB*/*FecB* had 0.74 more lambs (*p* < 0.01) than those with genotype *FecB+*/*FecB+* [7]. Besides, the Kalehkoohi sheep with genotypes BB and B+ had 0.52 and 0.35 lambs, more than the homozygous wild-type, respectively [41]. The above evidence indicated that *FecB* gene was closely related to sheep prolificacy. Therefore, this study focus on two sheep groups in terms of *FecB* BB and *FecB* B+ genotype. To our knowledge, this study represents the first systematical genome-wide analysis of pituitary lncRNAs in sheep and might provide valuable resources for searching functional lncRNAs associated with sheep fecundity.

In this study, we identified 19,672 lncRNAs and 27,291 coding transcripts. Previous studies showed that most of the lncRNAs were located near protein-coding genes [42,43,44], which means that the lncRNAs have synergetic relationships with mRNAs. In addition, lncRNAs and mRNAs were widely exists in all chromosomes of sheep, the location of a lncRNA may imply its diverse function. Notably, small proportion of lncRNAs and mRNAs were located in the mitochondria, which indicated that the LncRNAs and mRNAs might participate in biological functions in the cytoplasm. Notably, the proportion of lncRNAs and mRNAs in chromosome NC-019459.2, NC-019474.2 and NC-019460.2 were greater than those in other chromosomes, which could be explained by the close relationship between these three chromosome and pituitary function. Moreover, the sequence length and exon number of mRNAs and lncRNAs in sheep pituitary have a similar pattern with those in rat pituitary [24]. In addition, the exon size and ORF length of lncRNAs and mRNAs are mostly within 400 bp. These results not only showed the potential lncRNAs identified in this study were reliable but also suggested the specificity of pituitary tissue.

In the present study, we screened 57 DE lncRNA transcripts and 298 DE mRNAs in Hu sheep pituitary between high and low prolificacy sheep. Based on GO enrichment analysis, 298 DEGs were specifically enriched in pituitary function, hormone synthesis-related and reproduction process terms, such as *SCAMP1*, *AAK1*, *GRIK2*, *SP3*, *NCOA6* and *AHR* and so forth, which suggested that these genes might affect the fecundity of Hu sheep. The down-regulated genes *KCNT2*, *CACNB2*, *GRIK2* and *LOC101104054*, as well as a new up-regulated gene Ovis_aries_newGene_131644 were enriched in the transporter activity, which might play an important role in hormone synthesis.

Furthermore, KEGG enrichment analysis showed that 20 signaling pathways were related with reproduction like cAMP, Oxytocin, mTOR and MAPK signaling pathway. Previous study showed that increasing cAMP concentration could improve porcine cumulus maturation and subsequent in vitro fertilization [45]. M4 receptor-mediated down-regulation of cAMP production can inhibit the secretion of acetylcholine (*ACH*) [46] and affect the function of the pituitary. Other studies have shown that BMPs regulate steroidogenesis at a downstream of cAMP synthesis in human granulosa-like tumor cell line cells [47]. In addition, the NKB/NKBR system participates in the neuroendocrine control of fish reproduction [48]. Our results showed that *PDE3B*, *BDNF*, LOC101104054, *TAB1* and *BRAF* expression were significantly different, which means they might be considered as potential candidate genes for further study on pituitary function. Interestingly, *CDK17*, *RB1* and *LOC101112318* are at the core of the interaction networks. *RB1* gene is involved in cell proliferation and apoptosis [49] and was studied in pituitary tumors [50]. However, how these key genes cooperate with each other to exert their effects on pituitary functions remains largely unknown and needs further study.

This study indicated that DE lncRNAs and their target DE genes might play a determinative role in the biofunction of the sheep pituitary. Then, we constructed the lncRNA–target gene interaction networks by integrating DE lncRNAs, target genes and their co–regulatory relationships. According to the networks, lncRNA MSTRG.259847.2 is cis-acting on its target gene *SMAD2*, which may be considered an important regulator in pituitary function and reproduction. *SMAD2* had been reported to interact with growth differentiation factor 9 (*GDF9*), FSHβ [51] and affect FSH synthesis [52]. Moreover, *GDF9* was a well-known gene that affected fertility by increasing the number of ovulation [53]. In our study, knocking down LncRNA MSTRG.259847.2 in sheep pituitary cells was accompanied by decreased expression of *SMAD2*. This result indicated that *SMAD2* might play a role in pituitary function through its interaction with the lncRNA MSTRG.259847.2. Furthermore, the LH levels in the LncRNA MSTRG.259847.2 knocking down group was significantly lower than in the control groups, which indicated that *SMAD2* might affect pituitary function by influencing LH expression. *FecB* gene expression in sheep pituitary with different genotypes has been reported [54]. FSH and LH secreted in the pituitary could affect the development and maturation of follicles, then affect the ewes litter size. Previous reports showed that the serum FSH concentration of *FecBB* ewes was significantly higher than that of ++ ewes during specific physiological periods [55,56,57]. Furthermore, our study found that hormone levels of LH in FecBB Hu sheep was higher than FecB+ Hu sheep during estrus period (data not published). This result indicated FecB gene might affect sheep lambing number by affecting pituitary hormone secretion.

Furthermore, lncRNA MSTRG.236403.5 has a predicted role in regulating *NMB*, which is a highly conserved bombesin-related peptide found in mammals. Studies have shown that *NMB* plays critical roles in physiological/pathological processes in mammals [58,59,60]. Additionally, *NMB* was expressed in anterior pituitary cells [61]. Boughton and Patel et al. (2013) found that intracerebroventricular administration of *NMB* to adult male rats significantly increased LH levels. These results were interpreted as evidence of the regulatory role of *NMB* in pituitary function. Furthermore, in our study, we found lncRNA MSTRG.9440.3 target *GPR61*, *SORT1*, *SYPL2* and *PSMA5* in sheep pituitary gland. Previous studies suggested that *GPR61* has a different expression in the pre- and post-ovulation of bovine anterior pituitary [62]. Although the ligand(s) and functions of *GPR61* are not clear, it is known to associate with the Gs protein and stimulate extracellular regulated protein kinases (*ERK*) signaling in neurons [63,64]. It was also reported to regulate cAMP in hamster ovary cells [65]. *ERK* and cAMP pathways play an important role in GnRH-induced LH secretion in gonadotrophs [66,67,68]. These findings implied that *GPR61* might affect ovine pituitary function. Another DE lncRNA MSTRG.225589.1 was predicted to target *ACHE*, which is a serine protease that catalyzes the hydrolysis of Acetylcholin (*ACh*) [69]. The findings showed that *ACHE* might have indirect or direct stimulatory effect on GnRH/LH secretion [70]. Therefore, *ACHE* may be important for pituitary function. Our study indicates the potential importance of lncRNA MSTRG.225589.1 in regulating pituitary function.

At last, we constructed DE lncRNA-target gene-DEG regulated networks. The majority of the DEGs are enriched in localization and locomotion terms and a small number of genes are enriched in reproduction terms. This may due to many factors affecting the fecundity, such as heredity [53], environment [71,72], nutrition [73,74], physiology [75] and management [76]. The key function of the pituitary gland is to influence fecundity as a connecting link in the HPOA through hormones and other regulatory factors. Taken together, the DE lncRNAs identified in this study might cooperate with their target genes and DEGs to regulate pituitary functions.

## 5. Conclusions

In conclusion, this pituitary transcriptomic study reveals the differential regulation of lncRNAs and mRNAs related to prolificacy in different *FecB* genotyping sheep. We screened a set of lncRNAs and genes relating to pituitary function and reproduction. The lncRNAs identified in this study shared many properties with other mammalian lncRNAs. According to the GO and KEGG databases, the target genes of DE lncRNAs and DEGs were annotated with multiple biological processes associated with the pituitary. The lncRNA-gene transcriptional regulatory network generated in this study provides a valuable resource of candidate lncRNAs, which could be utilized in the exploration of functional lncRNAs in the pituitary. Furthermore, these differential mRNAs and lncRNAs expression profiles provide a valuable resource for the molecular mechanisms underlying the sheep prolificacy.

## Supplementary Materials

The following are available online at https://www.mdpi.com/2073-4425/10/2/157/s1. Table S1: Primers for qRT-PCR. Table S2: Alignment of statistical results of reads. Table S3: Top GO 20 Terms. Table S4: The list of DEGs enriched for reproduction and Function of the pituitary related GO terms. Table S5: The list of DEGs enriched for reproduction and Function of the pituitary related KEGG pathway. Table S6: The list of DEGs enriched for network of the pituitary.

## References

[1] Mulsant P., Lecerf F., Fabre S., Schibler L., Monget P., Lanneluc I., Pisselet C., Riquet J., Monniaux D., Callebaut I. (2001). Mutation in Bone Morphogenetic Protein Receptor-IB is Associated with Increased Ovulation Rate in Booroola Mérino Ewes. Proc. Natl. Acad. Sci. USA.

[2] Wilson T., Wu X.Y., Juengel J.L., Ross I.K., Lumsden J.M., Lord E.A., Dodds K.G., Walling G.A., Mcewan J.C., O’Connell A.R. (2001). Highly Prolific Booroola Sheep Have a Mutation in the Intracellular Kinase Domain of Bone Morphogenetic Protein IB Receptor (ALK-6) That Is Expressed in Both Oocytes and Granulosa Cells1. Biol. Reprod..

[3] Souza C.J., Macdougall C., Macdougall C., Campbell B.K., Mcneilly A.S., Baird D.T. (2001). The Booroola (FecB) phenotype is associated with a mutation in the bone morphogenetic receptor type 1 B (BMPR1B) gene. J. Endocrinol..

[4] Davis G.H., Balakrishnan L., Ross I.K., Wilson T., Galloway S.M., Lumsden B.M., Hanrahan J.P., Mullen M., Mao X.Z., Wang G.L. (2006). Investigation of the Booroola (FecB) and Inverdale (FecX(I)) mutations in 21 prolific breeds and strains of sheep sampled in 13 countries. Anim. Reprod. Sci..

[5] Chu M.X., Liu Z.H., Jiao C.L., He Y.Q., Fang L., Ye S.C., Chen G.H., Wang J.Y. (2007). Mutations in BMPR-IB and BMP-15 genes are associated with litter size in Small Tailed Han sheep (Ovis aries). J. Anim. Sci..

[6] Fogarty N.M. (2009). A review of the effects of the Booroola gene (FecB) on sheep production. Small Rumin. Res..

[7] Wang W.M., Liu S.J., Li F.D., Pan X.Y., Li C., Zhang X.X., Ma Y.J., La Y.F., Xi R., Li T.F. (2015). Polymorphisms of the Ovine BMPR-IB, BMP-15 and FSHR and Their Associations with Litter Size in Two Chinese Indigenous Sheep Breeds. Int. J. Mol. Sci..

[8] Esteller M. (2011). Non-coding RNAs in human disease. Nat. Rev. Genet..

[9] Hombach S., Kretz M. (2016). Non-coding RNAs: Classification, Biology and Functioning. Adv. Exp. Med. Biol..

[10] Fatica A., Bozzoni I. (2014). Long non-coding RNAs: New players in cell differentiation and development. Nat Rev. Genet..

[11] Abdelmohsen K., Panda A., Kang M.J., Xu J., Selimyan R., Yoon J.H., Martindale J.L., De S., Wood W.H., Becker K.G. (2013). Senescence-associated lncRNAs: Senescence-associated long noncoding RNAs. Aging Cell.

[12] Arun G., Akhade V.S., Donakonda S. (2012). mrhl RNA, a Long Noncoding RNA, Negatively Regulates Wnt Signaling through Its Protein Partner Ddx5/p68 in Mouse Spermatogonial Cells. Mol. Cell. Biol..

[13] Ghosal S., Das S., Chakrabarti J. (2013). Long noncoding RNAs: New players in the molecular mechanism for maintenance and differentiation of pluripotent stem cells. Stem Cells Dev..

[14] Mulvey B.B., Olcese U., Cabrera J.R., Horabin J.I. (2014). An interactive network of long non-coding RNAs facilitates the Drosophila sex determination decision. Biochim. Biophys. Acta.

[15] Hansen T.B., Jensen T.I., Clausen B.H., Bramsen J.B., Finsen B., Damgaard C.K., Kjems J. (2013). Natural RNA circles function as efficient microRNA sponges. Nature.

[16] Li W., Notani D., Ma Q., Tanasa B., Nunez E., Chen A.Y., Merkurjev D., Zhang J., Ohgi K., Song X. (2013). Functional roles of enhancer RNAs for oestrogen-dependent transcriptional activation. Nature.

[17] Mau M., Corral J.M., Vogel H., Melzer M., Fuchs J., Kuhlmann M., de Storme N., Geelen D., Sharbel T.F. (2013). The Conserved Chimeric Transcript UPGRADE2 Is Associated with Unreduced Pollen Formation and Is Exclusively Found in Apomictic Boechera Species. Plant. Physiol..

[18] Gao W.L., Liu M., Yang Y.Y., Yang H.X., Liao Q.P., Bai Y., Li Y.X., Li D., Peng C., Wang Y.L. (2012). The imprinted H19 gene regulates human placental trophoblast cell proliferation via encoding miR-675 that targets Nodal Modulator 1 (NOMO1). RNA Biol..

[19] Keniry A., Oxley D., Monnier P., Kyba M., Dandolo L., Smits G., Reik W. (2012). The H19 lincRNA is a developmental reservoir of miR-675 that suppresses growth and Igf1r. Nat. Cell Biol..

[20] Nemoto T., Mano A., Shibasaki T. (2012). Increased expression of miR-325-3p by urocortin 2 and its involvement in stress-induced suppression of LH secretion in rat pituitary. Am. J. Physiol. Endocrinol. Metab..

[21] Schang A.L., Granger A., Querat B., Bleux C., Cohen-Tannoudji J., Laverriere J.N. (2013). GATA2-induced silencing and LIM-homeodomain protein-induced activation are mediated by a bi-functional response element in the rat GnRH receptor gene. Mol. Endocrinol..

[22] Ueharu H., Higuchi M., Nishimura N., Yoshida S., Shibuya S., Sensui K., Kato T., Kato Y. (2014). Expression of Kruppel-like factor 6, KLF6, in rat pituitary stem/progenitor cells and its regulation of the PRRX2 gene. J. Reprod. Dev..

[23] Yuan B., Han D.X., Dai L.S., Gao Y., Ding Y., Yu X.F., Chen J., Jiang H., Chen C.Z., Zhang J.B. (2015). A comprehensive expression profile of micrornas in rat’s pituitary. Int. J. Clin. Exp. Med..

[24] Han D.X., Sun X.L., Fu Y., Wang C.J., Liu J.B., Jiang H., Gao Y., Chen C.Z., Yuan B., Zhang J.B. (2017). Identification of long non-coding RNAs in the immature and mature rat anterior pituitary. Sci. Rep..

[25] Huang J.L., Cao S.W., Ou Q.S., Yang B., Zheng S.H., Tang J., Chen J., Hu Y.W., Zheng L., Wang Q. (2018). The long non-coding RNA PTTG3P promotes cell growth and metastasis via up-regulating PTTG1 and activating PI3K/AKT signaling in hepatocellular carcinoma. Mol. Cancer.

[26] Fu D., Zhang Y., Cui H. (2018). Long noncoding RNA CCAT2 is activated by E2F1 and exerts oncogenic properties by interacting with PTTG1 in pituitary adenomas. Am. J. Cancer Res..

[27] Leka-Emiri S., Chrousos G.P., Kanaka-Gantenbein C. (2017). The mystery of puberty initiation: Genetics and epigenetics of idiopathic central precocious puberty (ICPP). J. Endocrinol. Investig..

[28] Kong L., Zhang Y., Ye Z.Q., Liu X.Q., Zhao S.Q., Wei L., Gao G. (2007). CPC: Assess the protein-coding potential of transcripts using sequence features and support vector machine. Nucleic Acids Res..

[29] Sun L., Luo H.T., Bu D.C., Zhao G.G., Yu K.T., Zhang C.H., Liu Y.N., Chen R.S., Zhao Y. (2013). Utilizing sequence intrinsic composition to classify protein-coding and long non-coding transcripts. Nucleic Acids Res..

[30] Wang L., Park H.J., Dasari S., Wang S.Q., Kocher J.P., Li W. (2013). CPAT: Coding-Potential Assessment Tool using an alignment-free logistic regression model. Nucleic Acids Res..

[31] Finn R.D., Bateman A., Clements J., Coggill P., Eberhardt R.Y., Eddy S.R., Heger A., Hetherington K., Holm L., Mistry J. (2014). Pfam: The protein families database. Nucleic Acids Res..

[32] Anders S., Huber W. (2010). Differential expression analysis for sequence count data. Genome Biol..

[33] Mao X.Z., Cai T., Olyarchuk J.G., Wei L.P. (2005). Automated genome annotation and pathway identification using the KEGG Orthology (KO) as a controlled vocabulary. Bioinformatics.

[34] Saito R., Smoot M.E., Ono K., Ruscheinski J., Wang P.L., Lotia S., Pico A.R., Bader G.D., Ideker T. (2012). A travel guide to Cytoscape plugins. Nat. Methods.

[35] Yao X.L., Yang H., Zhang Y.L., Ren C.F., Nie H.T., Fan Y.X., Zhou W.J., Wang S.T., Feng X., Wang F. (2017). Characterization of GALNTL5 gene sequence and expression in ovine testes and sperm. Theriogenology.

[36] Di Bella G., Turco V.L., Potorti A.G., Rando R., Licata P., Dugo G. (2013). Statistical analysis of heavy metals in *Cerastoderma edule glaucum* and *Venerupis aurea laeta* from Ganzirri Lake, Messina (Italy). Environ. Monit. Assess.

[37] Feng X., Li F., Wang F., Zhang G., Pang J., Ren C., Zhang T., Yang H., Wang Z., Zhang Y. (2018). Genome-wide differential expression profiling of mRNAs and lncRNAs associated with prolificacy in Hu sheep. Biosci. Rep..

[38] Miao X., Luo Q., Zhao H., Qin X. (2016). Co-expression analysis and identification of fecundity-related long non-coding RNAs in sheep ovaries. Sci. Rep..

[39] Miao X.Y., Luo Q.M., Zhao H.J., Qin X.Y. (2016). Ovarian transcriptomic study reveals the differential regulation of miRNAs and lncRNAs related to fecundity in different sheep. Sci. Rep..

[40] Miao X., Qin Q.L. (2015). Genome-wide transcriptome analysis of mRNAs and microRNAs in Dorset and Small Tail Han sheep to explore the regulation of fecundity. Mol. Cell Endocrinol..

[41] Mahdavi M., Nanekarani S., Hosseini S.D. (2014). Mutation in BMPR-IB gene is associated with litter size in Iranian Kalehkoohi sheep. Anim. Reprod. Sci..

[42] Ulitsky I., Shkumatava A., Jan C.H., Sive H., Bartel D.P. (2011). Conserved function of lincRNAs in vertebrate embryonic development despite rapid sequence evolution. Cell.

[43] Nam J.W., Bartel D.P. (2012). Long noncoding RNAs in C. elegans. Genome Res..

[44] Vallot C., Huret C., Lesecque Y., Resch A., Oudrhiri N., Bennaceur-Griscelli A., Duret L., Rougeulle C. (2013). XACT, a long noncoding transcript coating the active X chromosome in human pluripotent cells. Nat. Genet..

[45] Appeltant R., Beek J., Vandenberghe L., Maes D., Van Soom A. (2015). Increasing the cAMP concentration during in vitro maturation of pig oocytes improves cumulus maturation and subsequent fertilization in vitro. Theriogenology.

[46] Zemkova H., Kucka M., Bjelobaba I., Tomic M., Stojilkovic S.S. (2013). Multiple cholinergic signaling pathways in pituitary gonadotrophs. Endocrinology.

[47] Miyoshi T., Otsuka F., Suzuki J., Takeda M., Inagaki K., Kano Y., Otani H., Mimura Y., Ogura T., Makino H. (2006). Mutual regulation of follicle-stimulating hormone signaling and bone morphogenetic protein system in human granulosa cells. Biol. Reprod..

[48] Biran J., Palevitch O., Ben-Dor S., Levavi-Sivan B. (2012). Neurokinin Bs and neurokinin B receptors in zebrafish-potential role in controlling fish reproduction. Proc. Natl. Acad. Sci. USA.

[49] Indovina P., Pentimalli F., Casini N., Vocca I., Giordano A. (2015). RB1 dual role in proliferation and apoptosis: Cell fate control and implications for cancer therapy. Oncotarget.

[50] Zhao H., Bauzon F., Bi E., Yu J.J., Fu H., Lu Z., Cui J., Jeon H., Zang X., Ye B.H. (2015). Substituting threonine 187 with alanine in p27Kip1 prevents pituitary tumorigenesis by two-hit loss of Rb1 and enhances humoral immunity in old age. J. Biol. Chem..

[51] Choi S.G., Wang Q., Jia J., Pincas H., Turgeon J.L., Sealfon S.C. (2014). Growth differentiation factor 9 (GDF9) forms an incoherent feed-forward loop modulating follicle-stimulating hormone β-subunit (FSHbeta) gene expression. J. Biol. Chem..

[52] Wang Y., Ho C.C., Bang E., Rejon C.A., Libasci V., Pertchenko P., Hebert T.E., Bernard D.J. (2014). Bone morphogenetic protein 2 stimulates noncanonical SMAD2/3 signaling via the BMP type 1A receptor in gonadotrope-like cells: Implications for FSH synthesis. Endocrinology.

[53] Abdoli R., Zamani P., Mirhoseini S.Z., Ghavi Hossein-Zadeh N., Nadri S. (2016). A review on prolificacy genes in sheep. Reprod. Domest. Anim..

[54] Tang J.S., Hu W.P., Di R., Liu Q.Y., Wang X.Y., Zhang X.S., Zhang J.L., Chu M.X. (2018). Expression Analysis of the Prolific Candidate Genes, BMPR1B, BMP15, and GDF9 in Small Tail Han Ewes with Three Fecundity (FecB Gene) Genotypes. Animals.

[55] Chowdhury K.M., Sukanta S., Bhuyan M.A., Ali S.M., Ishaque A.M., Hai M.A. (1993). Plasma LH and FSH levels in azoospermia and in normal male and female human subjects. Bangladesh Med. Res. Counc. Bull..

[56] Mcnatty K.P., Hudson N.L., Lun S., Heath D.A., Shaw L., Condell L., Phillips D.J., Clarke I.J. (1993). Gonadotrophin-releasing hormone and the control of ovulation rate by the FecB gene in Booroola ewes. J. Reprod. Fertil..

[57] Phillips D.J., Hudson N.L., Mcnatty K.P. (1993). Effects of ovariectomy and genotype on bioactive FSH in plasma and pituitary of Booroola ewes. J. Reprod. Fertil..

[58] Matusiak D., Glover S., Nathaniel R., Matkowskyj K., Yang J.X., Benya R.V. (2005). Neuromedin B and its receptor are mitogens in both normal and malignant epithelial cells lining the colon. Am. J. Physiol.-Gastr. L.

[59] Oliveira K.J., Ortiga-Carvalho T.M., Cabanelas A., Veiga M.A.L.C., Aoki K., Ohki-Hamazaki H., Wada K., Wada E., Pazos-Moura C.C. (2006). Disruption of neuromedin B receptor gene results in dysregulation of the pituitary-thyroid axis. J. Mol. Endocrinol..

[60] Saito H., Nakamachi T., Inoue K., Ikeda R., Kitamura K., Minamino N., Shioda S., Miyata A. (2013). Autocrine effects of neuromedin B stimulate the proliferation of rat primary osteoblasts. J. Endocrinol..

[61] Kameda H., Miyoshi H., Shimizu C., Nagai S., Nakamura A., Kondo T., Chida D., Atsumi T. (2014). Expression and Regulation of Neuromedin B in Pituitary Corticotrophs of Male Melanocortin 2 Receptor-Deficient Mice. Endocrinology.

[62] Pandey K., Mizukami Y., Watanabe K., Sakaguti S., Kadokawa H. (2017). Deep sequencing of the transcriptome in the anterior pituitary of heifers before and after ovulation. J. Vet. Med. Sci..

[63] Takeda S., Yamamoto A., Okada T., Matsumura E., Nose E., Kogure K., Kojima S., Haga T. (2003). Identification of surrogate ligands for orphan G protein-coupled receptors. Life Sci..

[64] Toyooka M., Tujii T., Takeda S. (2009). The N-Terminal Domain of GPR61, an Orphan G-Protein-Coupled Receptor, Is Essential for Its Constitutive Activity. J. Neurosci. Res..

[65] Martin A.L., Steurer M.A., Aronstam R.S. (2015). Constitutive Activity among Orphan Class-A G Protein Coupled Receptors. PLoS ONE.

[66] Nakamura U., Kadokawa H. (2015). The nonsteroidal mycoestrogen zearalenone and its five metabolites suppress LH secretion from the bovine anterior pituitary cells via the estradiol receptor GPR30 in vitro. Theriogenology.

[67] Nakamura U., Rudolf F.O., Pandey K., Kadokawa H. (2015). The non-steroidal mycoestrogen zeranol suppresses luteinizing hormone secretion from the anterior pituitary of cattle via the estradiol receptor GPR30 in a rapid, non-genomic manner. Anim. Reprod. Sci..

[68] Rudolf F.O., Kadokawa H. (2016). Cytoplasmic kinases downstream of GPR30 suppress gonadotropin-releasing hormone (GnRH)-induced luteinizing hormone secretion from bovine anterior pituitary cells. J. Reprod. Dev..

[69] Taylor P., Radic Z. (1994). The Cholinesterases—From Genes to Proteins. Annu. Rev. Pharm..

[70] Herman A.P., Krawczynska A., Bochenek J., Haziak K., Romanowicz K., Misztal T., Antushevich H., Herman A., Tomaszewska-Zaremba D. (2013). The effect of rivastigmine on the LPS-induced suppression of GnRH/LH secretion during the follicular phase of the estrous cycle in ewes. Anim. Reprod. Sci..

[71] Goff K.J., Notter D.R., Vanimisetti H.B., Knight J.W. (2014). Strategies for rapid rebreeding of lactating ewes in the spring. Anim. Int. J. Anim. Biosci..

[72] Sejian V., Maurya V.P., Prince L.L., Kumar D., Naqvi S.M. (2015). Effect of FecB status on the allometric measurements and reproductive performance of Garole x Malpura ewes under hot semi-arid environment. Trop. Anim. Health Prod..

[73] Robertson S.M., Clayton E.H., Morgan B., Friend M.A. (2015). Reproductive performance in ewes fed varying levels of cut lucerne pasture around conception. Anim. Reprod. Sci..

[74] Selvaraju S., Raju P., Rao S.B.N., Raghavendra S., Nandi S., Dineshkumar D., Thayakumar A., Parthipan S., Ravindra J.P. (2012). Evaluation of maize grain and polyunsaturated fatty acid (PUFA) as energy sources for breeding rams based on hormonal, sperm functional parameters and fertility. Reprod. Fert. Dev..

[75] Nieto C.A.R., Thompson A.N., Macleay C.A., Briegel J.R., Hedger M.P., Ferguson M.B., Martin G.B. (2014). Relationships among body composition, circulating concentrations of leptin and follistatin, and the onset of puberty and fertility in young female sheep. Anim. Reprod. Sci..

[76] Dobson H., Fergani C., Routly J.E., Smith R.F. (2012). Effects of stress on reproduction in ewes. Anim. Reprod. Sci..

